# Common Genetic Variants Associated with Sudden Cardiac Death: The FinSCDgen Study

**DOI:** 10.1371/journal.pone.0041675

**Published:** 2012-07-23

**Authors:** Annukka M. Lahtinen, Peter A. Noseworthy, Aki S. Havulinna, Antti Jula, Pekka J. Karhunen, Johannes Kettunen, Markus Perola, Kimmo Kontula, Christopher Newton-Cheh, Veikko Salomaa

**Affiliations:** 1 Research Programs Unit, Molecular Medicine and Department of Medicine, University of Helsinki, Helsinki, Finland; 2 Cardiovascular Research Center and Center for Human Genetic Research, Massachusetts General Hospital, Boston, Massachusetts, United States of America; 3 Program in Medical and Population Genetics, Broad Institute, Cambridge, Massachusetts, United States of America; 4 National Institute for Health and Welfare, Helsinki, Finland; 5 National Institute for Health and Welfare, Turku, Finland; 6 School of Medicine, University of Tampere and Centre for Laboratory Medicine, Tampere University Hospital, Tampere, Finland; 7 Institute of Molecular Medicine FIMM, University of Helsinki, Helsinki, Finland; University of Oxford, United Kingdom

## Abstract

**Background:**

Sudden cardiac death (SCD) accounts for up to half of cardiac mortality. The risk of SCD is heritable but the underlying genetic variants are largely unknown. We investigated whether common genetic variants predisposing to arrhythmia or related electrocardiographic phenotypes, including QT-interval prolongation, are associated with increased risk of SCD.

**Methodology/Principal Findings:**

We studied the association between 28 candidate SNPs and SCD in a meta-analysis of four population cohorts (FINRISK 1992, 1997, 2002 and Health 2000, n = 27,629) and two forensic autopsy series (The Helsinki Sudden Death Study and The Tampere Autopsy Study, n = 694). We also studied the association between established cardiovascular risk factors and SCD. Causes of death were reviewed using registry-based health and autopsy data. Cox regression and logistic regression models were adjusted for age, sex, and geographic region. The total number of SCDs was 716. Two novel SNPs were associated with SCD: *SCN5A* rs41312391 (relative risk [RR] 1.27 per minor T allele, 95% CI 1.11–1.45, *P* = 3.4×10^−4^) and rs2200733 in 4q25 (RR 1.28 per minor T allele, 95% CI 1.11–1.48, *P* = 7.9×10^−4^). We also replicated the associations for 9p21 (rs2383207, RR 1.13 per G allele, 95% CI 1.01–1.26, *P* = 0.036), as well as for male sex, systolic blood pressure, diabetes, cigarette smoking, low physical activity, coronary heart disease, and digoxin use (*P*<0.05).

**Conclusions/Significance:**

Two novel genetic variants, one in the cardiac sodium channel gene *SCN5A* and another at 4q25 previously associated with atrial fibrillation, are associated with SCD.

## Introduction

Sudden cardiac death (SCD) is a major public health problem, accounting for 180,000–250,000 deaths per year in the United States [1]. Up to half of cardiac deaths occur suddenly [1], [2], and in approximately half of SCD cases, death is the first clinical manifestation of cardiac disease [1]. Therefore, improved risk stratification is needed. Established risk factors for coronary heart disease (CHD) predispose to SCD, including: advanced age, male sex, elevated blood pressure or serum cholesterol, reduced pulmonary vital capacity, lack of physical activity, smoking, excessive alcohol consumption, high body mass index (BMI), diabetes, rapid heart rate, and electrocardiographic abnormalities [3]–[5]. In addition to the well established clinical risk factors, a family history of SCD confers additional risk [6], [7] but the genetic variants underlying the inherited risk component of SCD are largely unknown.

Rare mutations in potassium and sodium channel genes cause long QT syndrome (LQTS) 8–10, marked by delayed ventricular repolarization and increased risk of ventricular tachycardia and SCD. Common variants in these LQTS genes are associated with electrocardiographic QT prolongation [11], [12], which has been associated with SCD in the general population [13]. QT-interval prolongation predisposes the myocardium to early afterdepolarizations, which may trigger ventricular arrhythmias, ventricular fibrillation, and ultimately SCD [14]. Since QT interval is highly heritable [15], it may provide an intermediate, continuous trait suitable for exploring the genetics of SCD. Other potential candidate variants to influence SCD risk include common variants associated with electrocardiographic PR interval, prolongation of which has been shown to be associated with all-cause mortality [16], and common variants associated with nonfatal arrhythmia such as atrial fibrillation, which has been reported to predispose to SCD after acute myocardial infarction [17].

The present study investigated the role of common genetic variants with recently reported associations with arrhythmia-related phenotypes, including atrial fibrillation, QT interval and PR interval, as potential modifiers of SCD risk. We have previously reported a study focused principally on the genetic components of QT interval [18]. The previous study analyzed 14 QT-interval-associated single nucleotide polymorphisms (SNPs) in 6,808 individuals from Health 2000 (including the Mini-Finland sample), experiencing only 116 SCD events. The current study examined 28 SNPs in a total of 28,323 individuals, experiencing 716 SCD events, and identified two novel SNPs associated with increased risk of SCD. In addition, an analysis of cardiovascular risk factors associated with SCD was carried out in four population cohorts including a total of 27,629 individuals.

## Methods

### Ethics statement

All included studies were carried out in accordance with the Declaration of Helsinki and had appropriate ethical approvals from the Ethics Committee of the National Public Health Institute, the Epidemiology Ethics Committee of the Helsinki and Uusimaa hospital region, the Ethics Committee of the Department of Forensic Medicine, University of Helsinki, the Tampere University Hospital Ethics Committee, and/or the National Supervisory Authority for Welfare and Health (Valvira). Written informed consent was obtained from the participants of the FINRISK and Health 2000 population studies. In the case of forensic samples included in The Helsinki Sudden Death Study (HSDS) [19] and The Tampere Autopsy Study (TASTY) [20], a complete medicolegal autopsy has to be performed in Finland in all cases of unexpected out-of-hospital death of a person without a history of serious disease, or if the person may have died a non-natural death. This procedure does not need the permission of the relatives and can be also accomplished even if the relatives are against the autopsy. At the forensic autopsy, all necessary routine samples can be taken to find out the cause of death. In the case of scientific research, the study protocol has to be first approved by the ethics committee of the hospital and after that permission for taking study samples from forensic autopsies has to be obtained from the National Supervisory Authority for Welfare and Health (Valvira), without need to contact the relatives to obtain informed consent.

### Study materials

The study samples consisted of FINRISK 1992 (n = 6,051), FINRISK 1997 (n = 8,446), FINRISK 2002 (n = 8,648), and Health 2000 (n = 9,013, including the Mini-Finland sample, n = 985) recruited from the Finnish population, as well as of the HSDS (n = 297) and TASTY (n = 397) series of forensic autopsies. The clinical characteristics of the study samples are shown in Table 1 and Table S1. FINRISK is a series of population-based health examination surveys focused mainly on cardiovascular risk factors and carried out at 5-year intervals for the age group of 25–74 years [21]. Each FINRISK cohort was drawn independently of each other from the population register for each study area. The sampling was stratified by sex, 10-year age group and study area, so that the cell size was generally 250 persons. Because FINRISK samples are independent population samples drawn every five years from the same geographical areas, it is possible that the same individual by coincidence ends up participating in more than one FINRISK survey. On average, 0.8% of participants of a FINRISK cohort were included by chance in another FINRISK cohort. This percentage was considered low and therefore unlikely to bias the analyses. DNA was available from 21,229 FINRISK participants. The Health 2000 Study is a two-stage stratified cluster sample collected between 2000 and 2001 and representing the Finnish population aged ≥30 years [22]. The Mini-Finland Health Survey (n = 8,000) was initially conducted between 1978 and 1980 similarly to The Health 2000 Study [23]. Of the Mini-Finland participants, 985 participated in a follow-up study in 2001. Health 2000 and Mini-Finland cohorts (DNA available for 6,400 individuals) were pooled for analysis, adjusting for study cohort.

**Table 1:** Clinical characteristics of the study samples.

	Population cohorts				Autopsy studies	
	FINRISK 1992	FINRISK 1997	FINRISK 2002	Health 2000	HSDS	TASTY
N with DNA available	5345	7672	8212	6400	297	397
Source population	Finland (4 regions*)	Finland (5 regions†)	Finland (6 regions‡)	Finland (the whole population aged ≥30 years)	Finland (region of Helsinki)	Finland (region of Tampere)
Age at baseline, mean ± SD	44.3±11.3	48.4±13.4	48.0±13.1	53.0±13.0	52.2±9.5	60.5±13.0
Sex, male, %	46.3	49.7	46.6	45.9	100	68.5
N of SCD	129	178	75	112	117	105

HSDS  =  The Helsinki Sudden Death Study, SCD  =  sudden cardiac death, SD  =  standard deviation, TASTY  =  The Tampere Autopsy Study.

* Helsinki/Vantaa, Turku/Loimaa, North Karelia, and North Savo.

† Helsinki/Vantaa, Turku/Loimaa, North Karelia, North Savo, and Oulu province.

‡ Helsinki/Vantaa, Turku/Loimaa, North Karelia, North Savo, Oulu province, and Lapland.

HSDS is a series of consecutive forensic autopsies of men aged 35–69 who died out of hospital in Helsinki between 1991 and 1992. The source sample comprised all out-of-hospital deaths of previously healthy men or those who had not seen a doctor for more than one year, excluding decomposed or mutilated bodies [19]. TASTY included consecutive medico-legal autopsies of men and women ≤97 years of age performed in the city of Tampere from 2002 to 2004 [20]. Subjects aged >80 or <25 years were excluded from the current study. In the population cohorts, subjects who became 80 years old during the follow-up were censored at their 80^th^ birthday. The age cut-offs were applied in order to increase the specificity of the SCD classification and to increase the homogeneity between the population cohorts collected from the general adult population and the series of forensic autopsies. Electrocardiographic data were available only from The Health 2000 Study and has been described in a separate report [18].

### Adjudication of causes of death

The causes of death were adjudicated using data from FINRISK and Health 2000 baseline investigations and prospective clinical information from the four national health care registries: the Causes of Death Registry, the Hospital Discharge Registry, the Drug Reimbursement Registry, and the Pharmacy Database (Methods S1). In the combined population cohorts, 65.1% of all SCDs and 75.1% of out-of-hospital SCDs underwent autopsy. In the entire study (including the autopsy series), 75.9% of SCDs underwent autopsy. The follow-up extended until the end of 2008, was based on personal ID codes, and covered 100% of all study subjects living in Finland (in FINRISK, 99.6% of all participants). In total, there were 279,758 person-years of follow-up (Table S1).

All deaths occurring in the study cohorts were classified as probable, possible or unlikely SCD, or death of unknown cause, by two independent physician reviewers evaluating all cohort baseline data and prospective follow-up data from the four national registries. In cases of disagreement, two additional physicians reviewed the data independently, and final adjudication was achieved by consensus of all four physicians. Possible and probable SCDs were pooled for the main analyses. In sensitivity analyses, only probable SCDs were included. For a detailed description of case adjudication, see Supporting Information (Methods S1 and Table S2).

Sensitivity analyses were carried out using cardiac deaths or all-cause deaths as the outcome. In these analyses, cardiac deaths were defined as: ICD-10 I00–I52, R96, R98, or R99 (ICD-9 390–429, 798, 799) either as the underlying or the immediate cause of death in the Causes of Death Registry.

The definitions of prevalent CHD and heart failure are described in Methods S1. Analysis of use of QT-prolonging medication was based on listings from the website http://www.qtdrugs.org (Table S3). The category of QT-shortening medication included only digoxin.

### Genotyping and expression analysis

A total of 30 common (minor allele frequency >1%) SNPs were selected based on recently reported associations with SCD-related phenotypes, including atrial fibrillation, QT interval and PR interval (Table S4). Genomic DNA was genotyped using the Sequenom iPLEX Gold assay (MALDI-TOF mass spectrometry, MassARRAY Analyzer Compact, Sequenom Inc., San Diego, CA, USA). For SNPs, quality control thresholds applied were ≥90% genotyping success in the combined population materials and Hardy-Weinberg equilibrium *P*>0.002 in each study sample. Of the 30 genotyped SNPs, 28 passed quality control filters; rs11756440 and rs2074518 were excluded (Table S5). For each study subject, ≥80% genotyping success across all passing SNPs was required for each genotyping pool separately.

Expression analysis of two significant SNPs, rs41312391 and rs2200733, was performed on RNA samples extracted from peripheral blood of 510 unrelated individuals aged 25–74 years (mean 51.9±13.7 years, females 53.8%) from the Helsinki region using Illumina HumanHT-12 Expression BeadChips (Illumina Inc., San Diego, CA, USA) as described previously [24]. Individuals used in the gene expression analysis represent the general Finnish population and were not selected for any trait.

### Statistical analyses

The QT-interval genotype score (QT_score_), which aggregates the information from 12 QT-interval-associated SNPs (Table S6), was calculated for each individual as described previously [18]. In FINRISK and Health 2000 cohorts, a Cox proportional hazards regression model was applied for analyzing the association between genotype and time to SCD. Age was used as the time scale in the Cox model and primary adjustments included sex and geographic region (East vs. West). In the second model, additional adjustments were applied for established cardiovascular risk factors (high-density lipoprotein (HDL)-total cholesterol concentration ratio, systolic blood pressure, prevalent diabetes, BMI, current and former smoking, moderate/high vs. low leisure-time physical activity) and prevalent CHD. Models 3 and 4 adjusted for all covariates in model 2 as well as for QT-prolonging and QT-shortening drug use in model 3 and prevalent heart failure in model 4. An additional model for rs2200733 also adjusted for atrial fibrillation (Minnesota codes 8.3.1 and 8.3.3) at baseline in the Health 2000 cohort in addition to the covariates in model 2. In the forensic autopsy series, the association between genotype and cause of death (SCD vs. unlikely SCD) was analyzed using logistic regression adjusting for age at death and sex. An additive genotypic model (genotypes coded as 0, 1, 2) was used in all analyses. Similar analyses were also performed for probable SCDs and all-cause and cardiac mortality adjusting for sex and geographic region. The association between baseline cardiovascular risk factors (see above) and time to SCD was investigated with a multivariate model in the population cohorts using Cox proportional hazards regression.

Analyses were performed with R version 2.11 (“survival” package) [25]. Inverse variance-weighted, fixed-effects meta-analysis (“meta” package) was performed for pooling of risk estimates. Heterogeneity between studies was assessed with I^2^ statistic [26]. When significant heterogeneity occurred (I^2^>0.5), random-effects meta-analysis was applied. The Bonferroni-corrected significance threshold for 25 independent tests (*r* ^2^<0.5) was 0.002 (*P* = 0.05/25). In secondary analyses, *P*<0.05 was considered statistically significant. All tests were two-sided. The power to observe a hazard ratio of 1.30 in the Cox regression analyses in the prospective population cohorts (n = 27,629) was 99.1%. For 1-ms change in the linear QT_score_, the corresponding analyses had 80% power to detect a hazard ratio ≥1.031 and 90% power to detect a hazard ratio ≥1.034.

## Results

### Cardiovascular risk factors and SCD

Male gender, higher systolic blood pressure, prevalent diabetes, current and former cigarette smoking, and Eastern Finnish residency all increased the risk of SCD in the meta-analysis of FINRISK and Health 2000 population cohorts, whereas increased leisure-time physical activity reduced the risk (Table 2). Prevalent CHD and digoxin use were associated with elevated risk of SCD, but the use of QT-prolonging drugs was not significantly associated with SCD risk (Table 2).

**Table 2:** Cardiovascular risk factors associated with SCD in a multivariate model in four population-based cohorts.

	Population cohorts, HR (95% CI)				Fixed-effects meta-analysis		
	FINRISK 1992	FINRISK 1997	FINRISK 2002	Health 2000	I^2^	HR (95% CI)	*P*-value
Sex, male	3.37 (2.24–5.07)	2.34 (1.56–3.52)	3.12 (1.70–5.74)	2.59 (1.62–4.13)	0.00	2.79 (2.23–3.51)	<2.2×10^−16^
Geographic region, East	1.43 (1.05–1.95)	1.06 (0.78–1.44)	1.60 (0.92–2.79)	1.19 (0.80–1.76)	0.00	1.26 (1.05–1.51)	0.01
HDL/TC ratio	1.22 (0.11–13.4)	0.08 (0.01–1.08)	0.93 (0.04–23.6)	1.27 (0.08–21.1)	0.00	0.56 (0.15–2.16)	0.40
Systolic BP per 10 mmHg	1.17 (1.08–1.26)	1.06 (0.98–1.13)	1.12 (1.02–1.24)	1.06 (0.97–1.16)	0.35	1.10 (1.06–1.15)	4.8×10^−6^
Prevalent diabetes	2.26 (1.49–3.44)	1.59 (1.07–2.39)	1.57 (0.85–2.88)	1.49 (0.94–2.37)	0.00	1.74 (1.38–2.18)	2.2×10^−6^
BMI per kg/m^2^	1.05 (1.02–1.09)	1.01 (0.97–1.04)	1.04 (0.99–1.09)	0.97 (0.92–1.01)	0.71*	1.02 (0.98–1.06)	0.38
Current smoker	2.70 (1.85–3.94)	2.78 (1.89–4.09)	5.48 (3.04–9.86)	4.83 (2.84–8.22)	0.55*	3.55 (2.52–5.00)	3.9×10^−13^
Former smoker	1.21 (0.79–1.84)	1.28 (0.86–1.90)	1.08 (0.56–2.09)	2.36 (1.38–4.02)	0.39	1.39 (1.10–1.76)	0.007
Physical activity†	0.56 (0.41–0.78)	0.58 (0.42–0.80)	0.42 (0.26–0.66)	0.45 (0.31–0.65)	0.00	0.51 (0.43–0.62)	6.7×10^−13^
Prevalent CHD	4.77 (3.20–7.13)	2.70 (1.77–4.11)	3.56 (2.06–6.17)	5.10 (3.38–7.72)	0.47	3.99 (3.20–4.96)	<2.2×10^−16^
QT-prolonging drug	NA	0.76 (0.40–1.42)	1.29 (0.58–2.84)	0.85 (0.46–1.55)	0.00	0.90 (0.61–1.31)	0.57
Digoxin	NA	3.33 (1.98–5.60)	2.12 (0.75–6.01)	2.68 (1.41–5.08)	0.00	2.91 (2.00–4.24)	2.7×10^−8^

BMI  =  body mass index, BP  =  blood pressure, CHD  =  coronary heart disease, CI  =  confidence interval, HDL  =  high-density lipoprotein cholesterol, HR  =  hazard ratio (in Cox regression), I^2^  =  measure of heterogeneity between studies, SCD  =  sudden cardiac death, TC  =  total cholesterol.

* Random-effects meta-analysis was used because of significant heterogeneity between studies.

† Leisure-time physical activity (moderate/high vs. low).

### Common genetic variants associated with SCD

**Figure 1:**
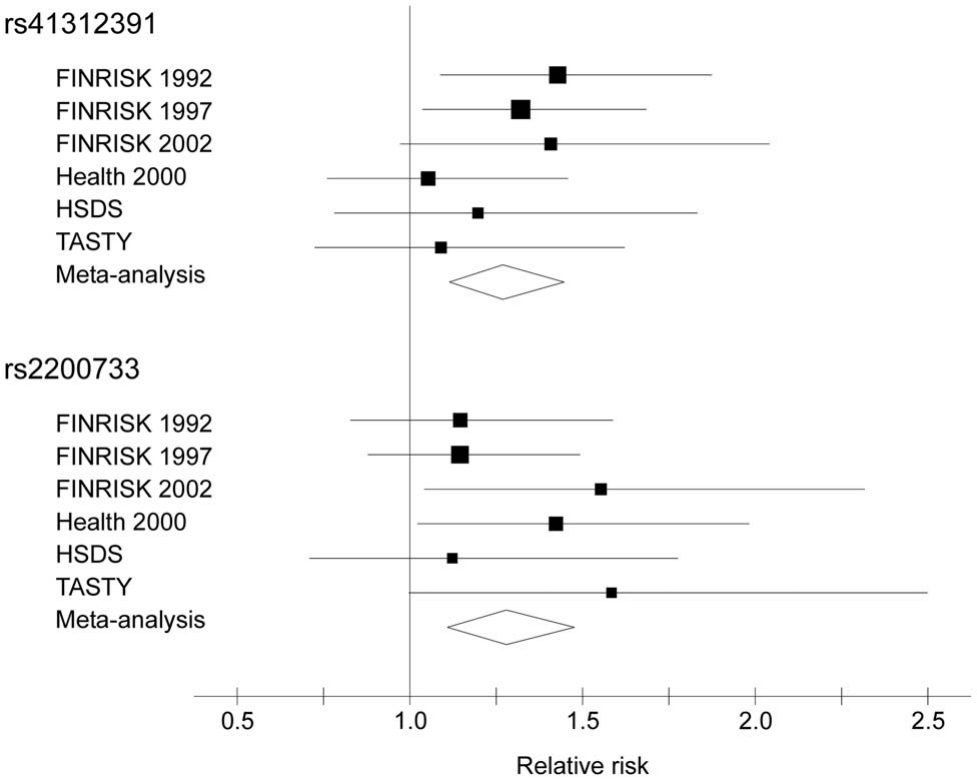
Forest plot of SCD risk ratios for rs41312391 and rs2200733. The figure shows point estimates for relative SCD risk (squares, area proportional to the inverse-variance weight) and 95% confidence intervals (horizontal lines) in all six cohorts, adjusting for sex and geographic region. The lateral tips of the diamonds indicate the 95% confidence intervals for the relative risk and the widest point of the diamond the relative risk estimate in the meta-analysis. HSDS  =  The Helsinki Sudden Death Study, SCD  =  sudden cardiac death, TASTY  =  The Tampere Autopsy Study.

Two SNPs were significantly associated with risk of SCD after Bonferroni correction: *SCN5A* rs41312391 (relative risk [RR] 1.27, 95% confidence interval [CI] 1.11–1.45, *P* = 3.4×10^−4^) and rs2200733 in 4q25 upstream of the *PITX2* gene (RR 1.28, 95% CI 1.11–1.48, *P* = 7.9×10^−4^) (Figure 1 and Table 3). The association results of these two SNPs were consistent among the different study cohorts (I^2^ = 0.00). In a sensitivity analysis restricting the cases to probable SCDs, the RR estimates remained similar: 1.28 (95% CI 1.11–1.48, *P* = 6×10^−4^) for rs41312391 and 1.27 (95% CI 1.08–1.49, *P* = 0.003) for rs2200733, implicating that inclusion of possible SCDs into the phenotype did not bias the results. In the expression analysis, the minor allele of rs41312391 was associated with increased expression of *WDR48* (*P* = 0.037) and the minor allele of rs2200733 with increased expression of *PITX2* (*P* = 0.013). The previously detected SCD association for rs2383207 in 9p21 [27] was replicated (RR 1.13, 95% CI 1.01–1.26, *P* = 0.036). None of the 28 SNPs was associated with all-cause mortality, but rs2200733 was associated with cardiac mortality (Table S7). The linear QT_score_ was not associated with SCD (*P* = 0.61) in the meta-analysis (Table S8).

**Table 3:** Results of the SCD meta-analysis for the two significant SNPs.

SNP	Model	Population cohorts, HR (95% CI)				Autopsy studies, OR (95% CI)		Fixed-effects meta-analysis		
		FINRISK 1992	FINRISK 1997	FINRISK 2002	Health 2000	HSDS	TASTY	I^2^	RR (95% CI)	*P*-value
rs41312391	1	1.43 (1.09–1.87)	1.32 (1.04–1.68)	1.41 (0.97–2.04)	1.05 (0.76–1.46)	1.20 (0.78–1.83)	1.09 (0.72–1.62)	0.00	1.27 (1.11–1.45)	3.4×10^−4^
rs41312391	2	1.53 (1.16–2.01)	1.33 (1.03–1.73)	1.44 (0.99–2.08)	1.07 (0.76–1.50)	NA	NA	0.00	1.35 (1.16–1.57)	1.2×10^−4^
rs2200733	1	1.15 (0.83–1.59)	1.15 (0.88–1.49)	1.55 (1.04–2.32)	1.42 (1.02–1.98)	1.12 (0.71–1.78)	1.58 (1.00–2.50)	0.00	1.28 (1.11–1.48)	7.9×10^−4^
rs2200733	2	1.06 (0.77–1.47)	1.23 (0.93–1.63)	1.67 (1.11–2.50)	1.30 (0.92–1.82)	NA	NA	0.00	1.26 (1.07–1.49)	0.006

Model 1: Age was used as the time scale and sex and geographic region were adjusted for. Model 2: Sex, geographic region, HDL-total cholesterol ratio, systolic blood pressure, prevalent diabetes, BMI, current and former smoking status, physical activity, and prevalent CHD were adjusted for. BMI  =  body mass index, CHD  =  coronary heart disease, CI  =  confidence interval, HDL  =  high-density lipoprotein cholesterol, HR  =  hazard ratio (in Cox regression), HSDS  =  The Helsinki Sudden Death Study, I^2^  =  measure of heterogeneity between studies, OR  =  odds ratio (in logistic regression), RR  =  relative risk, SCD  =  sudden cardiac death, SNP  =  single nucleotide polymorphism, TASTY  =  The Tampere Autopsy Study.

### SNP associations after adjustment for documented cardiovascular risk factors

Covariate adjustments in model 2 included sex, geographic region, cardiovascular risk factors (Table 2), and prevalent CHD. Use of QT-prolonging and QT-shortening medication were additionally adjusted for in model 3, and prevalent heart failure in model 4. Because these additional covariates were not available in HSDS and TASTY, models 2–4 could not be tested in these cohorts. *SCN5A* rs41312391 and 4q25 rs2200733 remained significant risk factors for SCD in all four models (Table 3 and Table S9). Adjustment for atrial fibrillation at baseline only slightly decreased the risk estimate of rs2200733 but the confidence intervals were wide (Table S9).

## Discussion

We studied the association of 28 common candidate SNPs with SCD in four large population-based cohorts and two forensic autopsy studies, altogether comprising 716 SCD cases among 28,323 individuals, and performed a meta-analysis combining the results of individual studies. Two SNPs were significantly associated with risk of SCD after correction for multiple testing: rs41312391 in the *SCN5A* sodium channel gene and rs2200733 in 4q25, which has been associated with atrial fibrillation [28]. In addition, this study replicates the association of rs2383207 in 9p21 with SCD [27].

Previously reported associations of SCD risk with male gender, higher systolic blood pressure, prevalent diabetes, current and former cigarette smoking, leisure-time physical activity, prevalent CHD, and Eastern Finnish residency were replicated [3]–[5], [19]. High BMI, high total cholesterol and low HDL cholesterol concentration, as well as the use of QT-prolonging drugs have been associated with increased risk of SCD in previous studies [3], [5], [29]. In the present study, BMI, the HDL/total cholesterol concentration ratio, and the use of QT-prolonging drugs were not significantly associated with risk of SCD. Interestingly, use of the QT-shortening drug digoxin was associated with an increased risk of SCD. This may be due to the fact that digoxin may have been prescribed to treat atrial fibrillation or heart failure, both of which are associated with SCD risk [17], [30]. Alternatively, digoxin use could be directly related to risk of SCD [31].

The *SCN5A* rs41312391 (IVS24+116G>A) minor allele has been associated with QT-interval prolongation in normal subjects in one study (n = 282, *P* = 0.04) [32] and QT-interval shortening in another (n = 396, *P* = 0.02) [33]. This SNP was not significantly associated with QT interval in the Health 2000 study (n = 4,802, β = −0.9 ms, *P* = 0.06). In the present study, the minor allele was found to have a frequency of 20% and to be associated with a 27% increased SCD risk. Adjusting for other cardiovascular risk factors and presence of CHD and heart failure at baseline did not reduce the SCD risk estimate, suggesting that the risk conferred by the allele may be independent of CHD and heart failure. *SCN5A* encodes the α-subunit of the cardiac sodium channel. Different mutations in *SCN5A* are known to cause congenital LQTS, Brugada syndrome, progressive cardiac conduction disease, sick sinus syndrome, dilated cardiomyopathy, and atrial fibrillation [34]. The expression analysis indicated that rs41312391 may change the expression level of a nearby gene, *WDR48*, which encodes a WD repeat-containing protein, a regulator of histone deubiquitinating complexes. However, *SCN5A* remains the more likely candidate gene for SCD due to its known function in myocardial repolarization and conduction processes that can result in SCD when deranged.

The minor T allele of rs2200733 at 4q25 has a population frequency of 16% and was associated with a 28% increased risk of SCD in our study, independent of the presence of CHD risk factors at baseline. It was also associated with a 28% increased risk of cardiac mortality, which implicates that it is also involved in non-sudden cardiac death. The same allele has been reported to predispose to atrial fibrillation in populations of both European and Chinese ancestry [28]. Our additional adjustment model suggests that the association of rs2200733 with SCD does not appear to be entirely explained by the increased risk of atrial fibrillation but may also involve other mechanisms, such as increased arrhythmia risk or abnormal cardiac function in general. However, a possibility of residual confounding also exists, since atrial fibrillation may not always have been captured by the baseline electrocardiogram. The expression analysis indicated that rs2200733 is associated with the expression of *PITX2*, the nearest gene in this chromosomal region. *PITX2* is a transcription factor known to direct the left-right asymmetry in cardiac development [35] and to contribute to sinoatrial node formation [36]. *PITX2* expression level is associated with structural and electrical changes in the atrial chambers, as well as expression of ion channel genes, including *SCN5A* [37]. Our results show that *PITX2* represents a novel candidate gene for both SCD and non-sudden cardiac death.

The minor G allele of rs2383207 on chromosome 9p21 has previously been associated with a 25% increased risk of myocardial infarction [38] and a 23% increased risk of SCD [27]. Our study replicates this association for SCD. The nearest genes in this 9p21 chromosomal region are the cyclin-dependent kinase inhibitor genes *CDKN2A* and *CDKN2B* as well as the *CDKN2B* antisense RNA gene. Deletion of the orthologous chromosomal region in mice leads to reduced expression of *CDKN2A* and *CDKN2B* as well as to excessive proliferation and diminished senescence of aortic smooth muscle cells [39]. Accordingly, it has been suggested that the variants in the 9p21 region predispose to CHD by affecting vascular remodeling [39].

Of the 28 candidate SNPs investigated in the present study, 25 did not show a statistically significant association with SCD. The power to identify a hazard ratio of >1.30 in the population cohorts was high (>99%) but this study cannot exclude the possibility of an association with a more modest effect size. Therefore, larger meta-analyses are needed to assess the role of candidate gene variants that may increase the risk of SCD by acting through arrhythmia-related intermediate phenotypes. However, this study was able to demonstrate the potential of identifying novel SCD-associated variants using suitable intermediate phenotypes for selection of candidate variants. When investigating the QT-prolonging variants in aggregate, the present study did not demonstrate an association between the linear QT_score_ and SCD, nor did it replicate the previously reported finding of an increased risk of SCD at the top compared to the median quintiles of QT_score_ [18]. Failure to detect an association could reflect a complex interaction between QT-prolonging and QT-shortening variants and risk of SCD or could be caused by inadequate power to detect a modest effect on SCD risk.

This study explored the association of a wide selection of candidate SNPs with SCD in a large sample collection of four population cohorts and two series of forensic autopsies, including a total of 716 SCD events. A strength of the present study is the availability of large prospective population cohorts enabling more precise risk estimates than case-control studies. In addition, prospective studies enable exploration of the effects of covariate adjustment. The long-term follow-up of the study subjects was comprehensive covering all subjects living in Finland, the autopsy rate of SCD cases was high (75.9%), and extensive information on the causes of death was gathered through several national registries. In Finland, autopsies are targeted on cases in which clinical information is insufficient to determine the cause of death. As the diagnoses in the death certificate are generally valid, including cases without autopsy [40], [41], the information obtained from the national registries as well as from the baseline investigations can be considered sufficient for reliable classification of causes of death. Finns represent a genetically isolated population with significant substructure due to founder effects and bottlenecks during the settlement history [42]. Significant East-West differences have previously been reported in the risk of SCD [19] and we replicated this finding. To account for population stratification in the present study, geographic region (East vs. West) was adjusted for in the statistical analyses of the population cohorts.

A large proportion of SCDs in Western countries are unwitnessed and therefore the presence of an underlying fatal arrhythmia, and the time between onset of symptoms and death cannot be reliably determined in a population-based study. The inclusion of individuals with a wide age range (25–80 years) increases the generalizability of the study but at the expense of increasing heterogeneity of the causes of death. Limitations of the study include also incomplete SNP information for constructing the QT_score_, missing covariate information in the forensic autopsy materials, and missing registry-based medication information before year 1995 (FINRISK 1992). Covariate information was available at baseline, which could be many years earlier than the date of death.

### Conclusions

We report two novel SNPs associated with increased risk of sudden cardiac death (SCD). In addition, our results replicate the association of a SNP in the chromosomal region 9p21 with SCD. Our study replicates the previously reported associations between well-known cardiovascular risk factors and risk of SCD. The novel findings of this study add to our understanding of the contributions of common genetic variants to SCD risk.

## Supporting Information

Methods S1 **Supporting information on data sources, classification of causes of death, and disease definitions.**
(PDF)

Table S1 **Follow-up time and clinical characteristics of the prospective study cohorts.**
(PDF)

Table S2 **Causes of death in each study cohort.**
(PDF)

Table S3 **QT-prolonging medication according to the website** http://www.qtdrugs.org/ **.**
(PDF)

Table S4 **SNPs selected for genotyping.**
(PDF)

Table S5 **SNP genotyping results in the different study samples.**
(PDF)

Table S6 **SNP information for QT_score_ calculation.**
(PDF)

Table S7 **Meta-analysis of all-cause and cardiac mortality.**
(PDF)

Table S8 **Association of QT_score_ with SCD.**
(PDF)

Table S9 **SCD meta-analysis results for all studied SNPs.**
(PDF)
