# Supplementary material for: Common Genetic Variants Associated with Sudden Cardiac Death: The FinSCDgen Study
Source: PLoS One. 2012 Jul 23;7(7):e41675. doi: 10.1371/journal.pone.0041675 (PMC3402479; doi:10.1371/journal.pone.0041675)
Supplement: Methods S1 — Supporting information on data sources, classification of causes of death, and disease definitions. (PDF) [file pone.0041675.s001.pdf]

Methods S1

Data sources

The Hospital Discharge Registry

Records of all hospitalizations in Finland have been collected in a nationwide registry maintained by the National Institute for Health and Welfare since 1967. The main diagnosis and up to three side diagnoses (both symptoms and cause) for each hospitalization are coded according to the International Classification of Diseases (ICD). Clinicians directly responsible for the care of the patient assign both the diagnoses and ICD codes for hospitalizations and logical checks for numerical data are done using a computer algorithm.

The Causes of Death Registry

The Causes of Death Registry is maintained by the Statistics Finland and catalogues the death certificate diagnoses (coded by ICD codes), which are assigned by the physician directly responsible for patient care at the time of death. The Causes of Death Registry lists the immediate, underlying, and up to four contributing causes of death according to the death certificate. The death certificate includes also a short summary of the final illness (completed by the physician), important clinical findings, diagnostic examinations, and findings of the autopsy if performed. Local authorities inspect the certificate for completeness prior to sending to Statistics Finland where uniform formatting is insured. If necessary, a forensic medicine specialist may clarify any unresolved death certificates.

The Drug Reimbursement Registry

A register of all medications prescribed for the treatment of certain severe, long-term illnesses in Finland (the Drug Reimbursement Registry) is maintained by the Finnish National Social Insurance Institution (KELA). A medical certificate based on examinations

performed by a specialist-level health care unit or issued by a specialist must be approved by a specialist physician working at KELA using predefined criteria for the diagnoses of the diseases before the patient can receive the right for drug reimbursement from KELA. As such, this database provides additional information about a patient's chronic diagnoses that can be used in cause of death adjudication.

Pharmacy data

Data on all drugs prescribed by a doctor and purchased by the patients are registered in a database maintained at KELA. Using this database, drugs can be identified using the Anatomical Therapeutic Chemical (ATC) classification codes.

Classification of causes of death

Probable SCDs included all deaths with a cardiac cause as the immediate or underlying cause of death, and without any other known cause of death than arrhythmia. Possible SCDs included deaths with a non-cardiac cause as the immediate or underlying cause of death, when cardiac disease was present and could reasonably have contributed to arrhythmia (e.g. unexpected death due to unwitnessed out-of-hospital aspiration in a patient with a prior myocardial infarction or cardiomyopathy). This group also included deaths that could have been arrhythmic based on circumstances (e.g. death of a driver in a motor vehicle crash or death while swimming in individuals with underlying cardiac disease or previous ventricular arrhythmia). Unlikely SCDs included deaths from a recognized medical cause unrelated to cardiac disease (e.g. cancer death, massive blood loss, sepsis, pulmonary embolism, stroke), deaths with a cardiac cause but apparently unrelated to lethal arrhythmia (e.g. myocardial rupture after myocardial infarction, endocarditis), and deaths of individuals who were hospitalized for >10 days prior to death. Deaths with insufficient data available to evaluate

their cause were classified within the category of unknown cause of death and were treated as unlikely SCDs.

Disease definitions

Definition of CHD

CHD was defined as either (a) diagnosis by the survey physician of previous myocardial infarction or coronary disease requiring surgical or percutaneous revascularization, (b) hospitalization with a main diagnosis of CHD (ICD-10 codes I200, I21, I22; or ICD-9 or ICD-8 codes 410, or 4110) or with a side diagnosis of MI (ICD-10 codes I21-I22; or ICD-8 or ICD-9 code 410), (c) diagnosis related to CHD (ICD-10 codes I20-I25, I46, R96, R98; or ICD-9 or ICD-8 codes 410-414, 798; not 7980A) as the immediate or underlying cause of death or diagnosis of MI (ICD-10 I21-I22; or ICD-8 or ICD-9 code 410) as the contributing cause of death, or (d) coronary revascularization based on The Finnish Hospital Discharge Registry. The validity of the CHD diagnoses in the Finnish national registries is generally good [1].

Definition of heart failure

Heart failure was defined as either (a) hospitalization with a diagnosis of heart failure (ICD-10 codes I50, I110, I130, I132; or ICD-9 codes 4029B, 4148, 428; or ICD-8 codes 42700, 42710, or 428), (b) prescribed medication of ATC codes C03CA01 or C03EB01, or (c) medication reimbursement with diagnosis codes I11.0, I13, I50, I97.1, or P29.0. In Health 2000 study, prevalent heart failure was defined as diagnosis by the survey physician, or diagnosis based on interview or registry follow-up.

Reference:

1. Pajunen P, Koukkunen H, Ketonen M, Jerkkola T, Immonen-Räihä P, et al. (2005) The validity of the Finnish Hospital Discharge Register and Causes of Death Register data on coronary heart disease. Eur J Cardiovasc Prev Rehabil 12: 132-137.
