# Supplementary material for: Common Genetic Variants Associated with Sudden Cardiac Death: The FinSCDgen Study
Source: PLoS One. 2012 Jul 23;7(7):e41675. doi: 10.1371/journal.pone.0041675 (PMC3402479; doi:10.1371/journal.pone.0041675)
Supplement: Table S1 — Follow-up time and clinical characteristics of the prospective study cohorts. (PDF) [file pone.0041675.s002.pdf]

Table S1. Follow-up time and clinical characteristics of the prospective study cohorts.

	FINRISK 1992	FINRISK 1997	FINRISK 2002	Health 2000
Follow-up time, years, median (IQR)	16.8 (0.10)	11.8 (0.11)	6.8 (0.11)	8.0 (0.31)
Person-years at risk	87991	87148	55888	48731
Geographic region, East, n (%)	2648 (48.9)	3073 (39.6)	2623 (31.6)	1936 (29.8)
HDL/TC ratio, mean ± SD	0.26 ± 0.079	0.26 ± 0.078	0.28 ± 0.085	0.23 ± 0.074
Systolic BP, mmHg, mean ± SD	135.5 ± 19.5	136.0 ± 20.0	135.0 ± 20.0	134.4 ± 20.6
Prevalent diabetes, n (%)	201 (3.7)	471 (6.1)	473 (5.7)	532 (8.2)
BMI, kg/m2, mean ± SD	26.1 ± 4.5	26.7 ± 4.6	26.9 ± 4.7	27.0 ± 4.7
Current smoker, n (%)	1512 (27.9)	1809 (23.3)	2142 (25.8)	1842 (28.4)
Former smoker, n (%)	1010 (18.6)	1727 (22.3)	1806 (21.8)	1337 (20.6)
Low physical activity, n (%)	1319 (24.6)	1754 (22.6)	1892 (22.8)	1619 (25.0)
Prevalent CHD, n (%)	68 (1.3)	201 (2.6)	213 (2.6)	269 (4.1)
QT-prolonging drug*, n (%)	NA[†]	405 (5.2)	474 (5.7)	551 (8.5)
Digoxin, n (%)	NA[†]	90 (1.2)	35 (0.4)	88 (1.4)

BMI = body mass index, BP = blood pressure, CHD = coronary heart disease, HDL = high-density lipoprotein cholesterol, IQR = interquartile range, SD = standard deviation, TC = total cholesterol.

*Any of the QT-prolonging drug classes (Table S3).

[†]Pharmacy data available only after 1995.
