# Supplementary material for: Common Genetic Variants Associated with Sudden Cardiac Death: The FinSCDgen Study
Source: PLoS One. 2012 Jul 23;7(7):e41675. doi: 10.1371/journal.pone.0041675 (PMC3402479; doi:10.1371/journal.pone.0041675)
Supplement: Table S2 — Causes of death in each study cohort. (PDF) [file pone.0041675.s003.pdf]

Table S2. Causes of death in each study cohort.

Cause of death	FINRISK 1992	FINRISK 1997	FINRISK 2002	Health 2000	HSDS	TASTY	All cohorts
Probable SCD	109	143	59	82	114	96	603
Possible SCD	20	35	16	30	3	9	113
Unlikely SCD	344	471	174	337	178	287	1791
Unknown	17	58	7	20	2	5	109
Total	490	707	256	469	297	397	2616

HSDS = The Helsinki Sudden Death Study, SCD = sudden cardiac death, TASTY = The Tampere Autopsy Study.
