# Supplementary material for: Common Genetic Variants Associated with Sudden Cardiac Death: The FinSCDgen Study
Source: PLoS One. 2012 Jul 23;7(7):e41675. doi: 10.1371/journal.pone.0041675 (PMC3402479; doi:10.1371/journal.pone.0041675)
Supplement: Table S3 — QT-prolonging medication according to the website http://www.qtdrugs.org/ . (PDF) [file pone.0041675.s004.pdf]

Table S3. QT-prolonging medication according to the website http://www.qtdrugs.org/.

Risk class	Generic name	ATC codes
Definite (revised 03/25/2008)	Amiodarone	C01BD01
	Arsenic trioxide	L01XX27
	Astemizole	R06AX11
	Bepridil	C08EA02
	Chloroquine	P01BA01
	Chlorpromazine	N05AA01
	Cisapride	A03FA02
	Clarithromycin	J01FA09, A02BD04, A02BD05, A02BD06
	Disopyramide	C01BA03
	Dofetilide	C01BD04
	Domperidone	A03FA03
	Droperidol	N05AD08, N01AX01
	Erythromycin	J01FA01
	Halofantrine	P01BX01
	Haloperidol	N05AD01
	Ibutilide	C01BD05
	Levomethadyl	N07BC03
	Mesoridazine	N05AC03
	Methadone	N07BC02, N02AC52
	Pentamidine	P01CX01
	Pimozide	N05AG02
	Probucol	C10AX02
	Procainamide	C01BA02
	Quinidine	C01BA01, C01BA51, C01BA71
	Sotalol	C07AA07, C07AA57, C07BA07
	Sparfloxacin	J01MA09
	Terfenadine	R06AX12
	Thioridazine	N05AC02
Possible (revised 04/15/2009)	Alfuzosin	G04CA01
	Amantadine	N04BB01
	Atazanavir	J05AE08
	Azithromycin	J01FA10
	Chloral hydrate	N05CC01, N05CC03
	Clozapine	N05AH02
	Dolasetron	A04AA04
	Felbamate	N03AX10
	Flecainide	C01BC04
	Foscarnet	J05AD01
	Fosphenytoin	N03AB05
	Gatifloxacin	J01MA16
	Gemifloxacin	J01MA15
	Granisetron	A04AA02
	Indapamide	C03BA11
	Isradipine	C08CA03
	Lapatinib	L01XE07
	Levofloxacin	J01MA12

	Lithium	N05AN01
	Moexipril/HCTZ	C09BA13
	Moxifloxacin	J01MA14
	Nicardipine	C08CA04
	Nilotinib	L01XE08
	Octreotide	H01CB02
	Ofloxacin	J01MA01
	Ondansetron	A04AA01
	Oxytocin	H01BB02, G02AC01, H01BB01
	Paliperidone	N05AX13
	Perflutren Lipid Microspheres	
	Quetiapine	N05AH04
	Ranolazine	C01EB18
	Risperidone	N05AX08
	Roxithromycin	J01FA06
	Sertindole	N05AE03
	Sunitinib	L01XE04
	Tacrolimus	L04AD02
	Tamoxifen	L02BA01
	Telithromycin	J01FA15
	Tizanidine	M03BX02
	Vardenafil	G04BE09
	Venlafaxine	N06AX16, N06AX23
	Voriconazole	J02AC03
	Ziprasidone	N05AE04
Conditional (revised 07/24/2009)	Amitriptyline	N06AA09, N06CA01
	Ciprofloxacin	J01MA02
	Citalopram	N06AB04, N06AB10
	Clomipramine	N06AA04
	Desipramine	N06AA01
	Diphenhydramine	R06AA02, R06AA52
	Doxepin	N06AA12
	Fluconazole	J02AC01
	Fluoxetine	N06AB03
	Galantamine	N06DA04
	Imipramine	N06AA02, N06AA03
	Itraconazole	J02AC02
	Ketoconazole	J02AB02
	Mexiletine	C01BB02
	Nortriptyline	N06AA10
	Paroxetine	N06AB05
	Protriptyline	N06AA11
	Sertraline	N06AB06
	Solifenacin	G04BD08
	Trimethoprim-Sulfa	J01EE01
	Trimipramine	N06AA06

ATC = Anatomical Therapeutic Chemical classification.
