# Supplementary material for: Common Genetic Variants Associated with Sudden Cardiac Death: The FinSCDgen Study
Source: PLoS One. 2012 Jul 23;7(7):e41675. doi: 10.1371/journal.pone.0041675 (PMC3402479; doi:10.1371/journal.pone.0041675)
Supplement: Table S4 — SNPs selected for genotyping. (PDF) [file pone.0041675.s005.pdf]

Table S4. SNPs selected for genotyping.

SNP	Chr	Position	Nearest gene	SNP function	Trait
rs846111	1p	6,201,957	*RNF207*	G603A	QT interval [1]
rs2880058	1q	160,281,256	*NOS1AP*	Upstream	QT interval [2,3]
rs12036340 (r^2 = 0.94 with rs12143842)	1q	160,282,364	*NOS1AP*	Upstream	QT interval [1,4]
rs12143842	1q	160,300,514	*NOS1AP*	Upstream	QT interval [1,4,5]
rs10919071	1q	167,366,107	*ATP1B1*	Intronic	QT interval [1,4]
rs1805126	3p	38,567,410	*SCN5A*	D1819D	QT, QRS and PR interval [1,6]
rs12053903	3p	38,568,397	*SCN5A*	Intronic	QT, QRS and PR interval [1,4,6]
rs41312391	3p	38,573,610	*SCN5A*	Intronic	QT interval [7,8]
rs3922844	3p	38,599,257	*SCN5A*	Intronic	PR interval [9]
rs6599219	3p	38,612,714	*SCN5A*	Intronic	PR interval*
rs7372712	3p	38,661,196	*SCN5A*	Intronic	PR interval*
rs2200733	4q	111,929,618	*PITX2*	Upstream	atrial fibrillation [10]
rs10033464	4q	111,940,210	*PITX2*	Upstream	atrial fibrillation [10]
rs1042714	5q	148,186,666	*ADRB2*	Q27E	SCD [11]
rs12210810	6q	118,759,897	*SLC35F1/PLN*	Downstream/ upstream	QT interval [1]
rs11756440 (r^2 = 1.0 with rs11756438)	6q	119,100,335	*C6orf204/PLN*	Intronic/ downstream	QT interval [4]
rs4725982	7q	150,268,796	*KCNH2*	Downstream	QT interval [4]
rs1805123	7q	150,276,467	*KCNH2*	K897T	QT interval [1,2,4]
rs3807375	7q	150,298,143	*KCNH2*	Intronic	QT interval [1,2,4,12]
rs2383207	9p	22,105,959	*CDKN2A/CDKN2B*	Upstream	MI, SCD [13,14]
rs2074238	11p	2,441,379	*KCNQ1*	Intronic	QT interval [4]
rs757092	11p	2,455,754	*KCNQ1*	Intronic	QT interval [1,15]
rs12576239	11p	2,458,895	*KCNQ1*	Intronic	QT interval [4]
rs10798	11p	2,826,741	*KCNQ1*	3´UTR	QT interval [16]
rs735951 (r^2 = 0.78 with rs8049607)	16p	11,601,037	*LITAF*	Upstream	QT interval [1,4]
rs37062	16q	57,124,739	*CNOT1*	Intronic	QT interval [4]
rs2074518	17q	30,348,495	*LIG3*	Intronic	QT interval [1,4]
rs17779747	17q	66,006,587	*KCNJ2*	Downstream	QT interval [1,4]
rs1805128	21q	34,743,550	*KCNE1*	D85N	QT interval [1,2,4]
rs727957	21q	34,801,942	*KCNE1*	Intronic	QT interval [1,15]

Chr = chromosome, MI = myocardial infarction, SCD = sudden cardiac death, SNP = single nucleotide polymorphism, UTR = untranslated region.

*Unpublished PR-interval candidate SNPs studied in the Framingham Heart Study.

References:

1. Pfeufer A, Sanna S, Arking DE, Müller M, Gateva V, et al. (2009) Common variants at ten loci modulate the QT interval duration in the QTSCD Study. Nat Genet 41: 407-414.
2. Marjamaa A, Newton-Cheh C, Porthan K, Reunanen A, Lahermo P, et al. (2009) Common candidate gene variants are associated with QT interval duration in the general population. J Intern Med 265: 448-458.
3. Marroni F, Pfeufer A, Aulchenko YS, Franklin CS, Isaacs A, et al. (2009) A genome-wide association scan of RR and QT interval duration in 3 European genetically isolated populations: the EUROSPAN project. Circ Cardiovasc Genet 2: 322-328.
4. Newton-Cheh C, Eijgelsheim M, Rice KM, de Bakker PI, Yin X, et al. (2009) Common variants at ten loci influence QT interval duration in the QTGEN Study. Nat Genet 41: 399-406.
5. Eijgelsheim M, Aarnoudse AL, Rivadeneira F, Kors JA, Witteman JC, et al. (2009) Identification of a common variant at the NOS1AP locus strongly associated to QT-interval duration. Hum Mol Genet 18: 347-357.
6. Holm H, Gudbjartsson DF, Arnar DO, Thorleifsson G, Thorgeirsson G, et al. (2010) Several common variants modulate heart rate, PR interval and QRS duration. Nat Genet 42: 117-122.
7. Aydin A, Bähring S, Dahm S, Guenther UP, Uhlmann R, et al. (2005) Single nucleotide polymorphism map of five long-QT genes. J Mol Med 83: 159-165.
8. Gouas L, Nicaud V, Chaouch S, Berthet M, Forhan A, et al. (2007) Confirmation of associations between ion channel gene SNPs and QTc interval duration in healthy subjects. Eur J Hum Genet 15: 974-979.
9. Smith JG, Magnani JW, Palmer C, Meng YA, Soliman EZ, et al. (2011) Genome-Wide Association Studies of the PR Interval in African Americans. PLoS Genet 7: e1001304.
10. Gudbjartsson DF, Arnar DO, Helgadottir A, Gretarsdottir S, Holm H, et al. (2007) Variants conferring risk of atrial fibrillation on chromosome 4q25. Nature 448: 353-357.
11. Sotoodehnia N, Siscovick DS, Vatta M, Psaty BM, Tracy RP, et al. (2006) Beta2-adrenergic receptor genetic variants and risk of sudden cardiac death. Circulation 113: 1842-1848.
12. Newton-Cheh C, Guo CY, Larson MG, Musone SL, Surti A, et al. (2007) Common genetic variation in KCNH2 is associated with QT interval duration: the Framingham Heart Study. Circulation 116: 1128-1136.
13. Helgadottir A, Thorleifsson G, Manolescu A, Gretarsdottir S, Blondal T, et al. (2007) A common variant on chromosome 9p21 affects the risk of myocardial infarction. Science 316: 1491-1493.
14. Newton-Cheh C, Cook NR, VanDenburgh M, Rimm EB, Ridker PM, et al. (2009) A common variant at 9p21 is associated with sudden and arrhythmic cardiac death. Circulation 120: 2062-2068.
15. Pfeufer A, Jalilzadeh S, Perz S, Mueller JC, Hinterseer M, et al. (2005) Common variants in myocardial ion channel genes modify the QT interval in the general population: results from the KORA study. Circ Res 96: 693-701.

16. Gouas L, Nicaud V, Berthet M, Forhan A, Tiret L, et al. (2005) Association of KCNQ1, KCNE1, KCNH2 and SCN5A polymorphisms with QTc interval length in a healthy population. Eur J Hum Genet 13: 1213-1222.
