# Supplementary material for: Common Genetic Variants Associated with Sudden Cardiac Death: The FinSCDgen Study
Source: PLoS One. 2012 Jul 23;7(7):e41675. doi: 10.1371/journal.pone.0041675 (PMC3402479; doi:10.1371/journal.pone.0041675)
Supplement: Table S5 — SNP genotyping results in the different study samples. (PDF) [file pone.0041675.s006.pdf]

Table S5. SNP genotyping results in the different study samples.

SNP	Coded allele	FINRISK and Health 2000 combined			FINRISK			Health 2000			HSDS			TASTY		
		AF	HWE *P*-value	Call rate	AF	HWE *P*-value	Call rate	AF	HWE *P*-value	Call rate	AF	HWE *P*-value	Call rate	AF	HWE *P*-value	Call rate
rs846111	C	0.26	0.58	1.00	0.26	0.66	1.00	0.25	0.73	1.00	0.25	0.44	1.00	0.27	0.08	1.00
rs2880058	G	0.36	0.98	1.00	0.36	0.81	1.00	0.35	0.60	0.99	0.34	0.74	1.00	0.34	0.12	1.00
rs12036340	G	0.25	0.19	0.98	0.25	0.24	0.98	0.24	0.52	0.99	0.24	0.58	1.00	0.25	0.21	1.00
rs12143842	T	0.25	0.60	1.00	0.25	0.42	1.00	0.24	0.69	1.00	0.24	0.52	1.00	0.25	0.16	0.99
rs10919071	G	0.11	0.36	0.99	0.10	0.43	0.99	0.11	0.64	0.99	0.11	0.74	1.00	0.12	0.28	0.99
rs1805126	G	0.44	0.17	1.00	0.44	0.12	1.00	0.43	0.95	0.99	0.39	0.57	1.00	0.42	0.08	1.00
rs12053903	C	0.41	0.18	0.99	0.41	0.38	1.00	0.41	0.23	0.99	0.37	0.40	1.00	0.38	0.22	1.00
rs41312391	T	0.20	0.02	0.98	0.20	0.003	0.99	0.20	0.68	0.97	0.18	0.19	1.00	0.19	0.93	1.00
rs3922844	T	0.26	0.82	1.00	0.26	0.54	1.00	0.26	0.52	1.00	0.24	0.84	0.95	0.27	0.12	0.94
rs6599219	G	0.37	0.60	1.00	0.37	0.74	1.00	0.36	0.64	0.99	0.37	0.12	1.00	0.35	0.06	1.00
rs7372712	T	0.16	0.15	1.00	0.16	0.11	1.00	0.16	0.94	1.00	0.14	0.30	1.00	0.19	0.55	1.00
rs2200733	T	0.16	0.02	0.96	0.16	0.04	0.96	0.16	0.28	0.98	0.17	0.99	0.99	0.13	0.88	0.98
rs10033464	T	0.17	0.92	0.92	0.17	0.75	0.91	0.16	0.68	0.96	0.16	0.48	0.97	0.15	0.96	0.86
rs1042714	G	0.38	0.30	1.00	0.38	0.03	1.00	0.38	0.07	0.99	0.35	0.69	1.00	0.36	0.90	1.00
rs12210810	C	0.03	0.10	1.00	0.03	0.30	1.00	0.03	0.13	1.00	0.03	0.66	1.00	0.03	0.61	1.00
rs11756440	A	0.46	$1.0x10^{-12}$	0.97	0.46	$6.6x10^{-10}$	0.97	0.47	$4.0x10^{-4}$	0.99	0.48	0.22	1.00	0.47	0.19	1.00
rs4725982	T	0.29	0.80	1.00	0.29	0.64	1.00	0.28	0.71	1.00	0.29	0.93	1.00	0.29	0.19	1.00
rs1805123	G	0.17	0.87	0.99	0.16	0.37	0.99	0.18	0.08	0.96	NA	NA	NA	NA	NA	NA
rs3807375	T	0.44	0.23	1.00	0.45	0.12	1.00	0.43	0.67	1.00	0.47	0.46	1.00	0.43	0.34	0.99
rs2383207	G	0.43	0.72	0.99	0.43	0.91	0.99	0.43	0.35	0.99	0.45	0.06	0.99	0.43	0.23	1.00
rs2074238	T	0.09	0.01	1.00	0.09	0.05	1.00	0.09	0.12	1.00	0.10	0.62	1.00	0.07	0.38	1.00

rs757092	G	0.36	0.59	0.99	0.36	0.56	1.00	0.36	0.95	0.98	0.38	0.59	0.97	0.32	0.83	0.89
rs12576239	T	0.16	0.80	1.00	0.16	0.44	1.00	0.16	0.38	1.00	0.16	0.95	1.00	0.17	0.94	1.00
rs10798	G	0.36	0.67	1.00	0.36	0.49	1.00	0.37	0.73	0.99	0.36	0.86	1.00	0.35	0.21	1.00
rs735951	A	0.42	0.37	1.00	0.42	0.83	1.00	0.42	0.15	0.99	0.45	0.27	1.00	0.42	1.00	0.99
rs37062	G	0.27	0.006	1.00	0.27	0.03	1.00	0.26	0.09	1.00	0.25	0.54	1.00	0.24	0.35	1.00
rs2074518	C	0.45	0.04	0.51	0.45	0.04	0.60	0.45	0.69	0.24	NA	NA	NA	NA	NA	NA
rs17779747	T	0.25	0.82	1.00	0.25	0.72	1.00	0.25	0.87	1.00	0.24	0.38	1.00	0.30	0.24	1.00
rs1805128	T	0.02	0.62	1.00	0.02	0.81	1.00	0.01	0.08	1.00	0.02	0.79	1.00	0.01	0.86	1.00
rs727957	T	0.17	0.30	1.00	0.17	0.34	1.00	0.17	0.67	1.00	0.18	0.32	1.00	0.18	0.87	1.00

Coded alleles refer to the allele coded 0,1,2 with reference to the positive strand of the reference sequence of the human genome—NCBI build 36.1. AF = coded allele frequency, HSDS = The Helsinki Sudden Death Study, HWE = Hardy-Weinberg equilibrium, NA = no genotype information available, SNP = single nucleotide polymorphism, TASTY = The Tampere Autopsy Study.
