# Supplementary material for: Common Genetic Variants Associated with Sudden Cardiac Death: The FinSCDgen Study
Source: PLoS One. 2012 Jul 23;7(7):e41675. doi: 10.1371/journal.pone.0041675 (PMC3402479; doi:10.1371/journal.pone.0041675)
Supplement: Table S6 — SNP information for QTscore calculation. (PDF) [file pone.0041675.s007.pdf]

Table S6. SNP information for QT_{score} calculation.

SNP	Chr	Position	Nearest gene	Coded allele	AF	Effect (ms)
rs846111	1p	6,201,957	*RNF207*	C	0.26	1.7
rs12143842	1q	160,300,514	*NOS1AP*	T	0.25	3.5
rs10919071	1q	167,366,107	*ATP1B1*	G	0.11	-1.8
rs12053903	3p	38,568,397	*SCN5A*	C	0.41	-1.1
rs12210810	6q	118,759,897	*SLC35F1 / PLN*	C	0.03	-2.8
rs4725982	7q	150,268,796	*KCNH2*	T	0.29	1.5
rs2074238	11p	2,441,379	*KCNQ1*	T	0.09	-5.0
rs12576239	11p	2,458,895	*KCNQ1*	T	0.16	1.9
rs735951	16p	11,601,037	*LITAF*	A	0.42	-1.2
rs37062	16q	57,124,739	*CNOT1*	G	0.27	-1.9
rs17779747	17q	66,006,587	*KCNJ2*	T	0.25	-1.1
rs1805128	21q	34,743,550	*KCNE1*	T	0.02	8.4

The QT_{score} SNPs were selected to represent independent signals with genome-wide significant association with QT interval [1,2]. The QT_{score} was calculated based on previously reported effect estimates [1,2]: QT_{score} = [(SNP1 allele copy number) x (SNP1 effect estimate in predicted ms)] + [(SNP2 allele copy number) x (SNP2 effect estimate in predicted ms)] + … through SNP12. For each individual, <40% of the QT_{score} SNPs were allowed to be missing. Missing QT_{score} SNP genotypes were imputed separately in each study using the formula: allele copy number = 2 x coded allele frequency [3]. Coded alleles refer to the allele coded 0,1,2 with reference to the positive strand of the reference sequence of the human genome—NCBI build 36.1. AF = coded allele frequency, Chr = chromosome, ms = millisecond, SNP = single nucleotide polymorphism.

References:

1. Newton-Cheh C, Eijgelsheim M, Rice KM, de Bakker PI, Yin X, et al. (2009) Common variants at ten loci influence QT interval duration in the QTGEN Study. Nat Genet 41: 399-406.
2. Pfeufer A, Sanna S, Arking DE, Müller M, Gateva V, et al. (2009) Common variants at ten loci modulate the QT interval duration in the QTSCD Study. Nat Genet 41: 407-414.
3. Purcell S, Neale B, Todd-Brown K, Thomas L, Ferreira MA, et al. (2007) PLINK: a tool set for whole-genome association and population-based linkage analyses. Am J Hum Genet 81: 559-575.
