# Supplementary material for: Common Genetic Variants Associated with Sudden Cardiac Death: The FinSCDgen Study
Source: PLoS One. 2012 Jul 23;7(7):e41675. doi: 10.1371/journal.pone.0041675 (PMC3402479; doi:10.1371/journal.pone.0041675)
Supplement: Table S7 — Meta-analysis of all-cause and cardiac mortality. (PDF) [file pone.0041675.s008.pdf]

Table S7. Meta-analysis of all-cause and cardiac mortality.

SNP	Population cohorts, HR (95% CI)*				Autopsy studies, OR (95% CI)†		Fixed-effects meta-analysis		
	FINRISK 1992	FINRISK 1997	FINRISK 2002	Health 2000	HSDS	TASTY	I^2	RR (95% CI)	*P*-value
**All-cause mortality**									
rs41312391	1.02 (0.88-1.19)	1.02 (0.89-1.18)	1.05 (0.84-1.32)	0.91 (0.75-1.10)	NA	NA	0.00	1.00 (0.92-1.09)	0.96
rs2200733	1.00 (0.84-1.19)	0.98 (0.85-1.14)	1.08 (0.85-1.37)	1.17 (0.97-1.42)	NA	NA	0.00	1.04 (0.95-1.14)	0.40
**Cardiac mortality**									
rs41312391	1.13 (0.88-1.46)	1.17 (0.93-1.47)	1.00 (0.66-1.53)	0.90 (0.65-1.26)	1.21 (0.79-1.84)	1.13 (0.77-1.65)	0.00	1.10 (0.97-1.25)	0.14
rs2200733	1.12 (0.82-1.53)	1.13 (0.88-1.45)	1.20 (0.77-1.87)	1.73 (1.28-2.34)	1.15 (0.73-1.81)	1.58 (1.02-2.45)	0.24	1.28 (1.12-1.47)	3.8×10^{-4}

CI = confidence interval, HR = hazard ratio (in Cox regression), HSDS = The Helsinki Sudden Death Study, I^2 = measure of heterogeneity between studies, OR = odds ratio (in logistic regression), RR = relative risk, SNP = single nucleotide polymorphism, TASTY = The Tampere Autopsy Study.

*Age was used as the time scale and sex and geographic region were adjusted for.

†Age at death and sex were adjusted for.
