# Supplementary material for: Common Genetic Variants Associated with Sudden Cardiac Death: The FinSCDgen Study
Source: PLoS One. 2012 Jul 23;7(7):e41675. doi: 10.1371/journal.pone.0041675 (PMC3402479; doi:10.1371/journal.pone.0041675)
Supplement: Table S8 — Association of QTscore with SCD. (PDF) [file pone.0041675.s009.pdf]

Table S8. Association of QT_{score} with SCD.

SNP	Population cohorts, HR (95% CI)*			Autopsy studies, OR (95% CI)[†]			Fixed-effects meta-analysis		
	FINRISK 1992	FINRISK 1997	FINRISK 2002	Health 2000	HSDS	TASTY	I^2	RR (95% CI)	*P*-value
Linear QT_{score}	0.99 (0.95-1.03)	1.01 (0.98-1.04)	0.98 (0.93-1.04)	1.03 (0.99-1.08)	0.99 (0.93-1.04)	1.04 (0.98-1.10)	0.00	1.01 (0.99-1.02)	0.61
QT_{score} 1st quintile[‡]	1.39 (0.79-2.45)	0.66 (0.42-1.04)	1.46 (0.69-3.08)	1.01 (0.51-2.03)	0.74 (0.35-1.57)	0.68 (0.32-1.42)	0.24	0.91 (0.70-1.17)	0.45
QT_{score} 2nd quintile[‡]	1.49 (0.85-2.62)	0.65 (0.41-1.03)	1.27 (0.59-2.71)	1.29 (0.67-2.47)	0.63 (0.29-1.35)	0.64 (0.30-1.33)	0.42	0.92 (0.71-1.18)	0.51
QT_{score} 4th quintile[‡]	1.27 (0.72-2.25)	0.92 (0.61-1.40)	1.47 (0.71-3.04)	1.78 (0.97-3.26)	0.71 (0.33-1.52)	1.04 (0.51-2.12)	0.05	1.13 (0.89-1.44)	0.32
QT_{score} 5th quintile[‡]	1.03 (0.57-1.86)	0.70 (0.44-1.11)	1.15 (0.53-2.53)	1.62 (0.87-3.02)	0.58 (0.26-1.24)	1.06 (0.52-2.13)	0.23	0.94 (0.73-1.22)	0.65

CI = confidence interval, HR = hazard ratio (in Cox regression), HSDS = The Helsinki Sudden Death Study, I^2 = measure of heterogeneity between studies, OR = odds ratio (in logistic regression), RR = relative risk, TASTY = The Tampere Autopsy Study.

*Age was used as time scale and sex and geographic region were adjusted for.

[†]Age at death and sex were adjusted for.

[‡]Third quintile used as reference.
