# Supplementary material for: Common Genetic Variants Associated with Sudden Cardiac Death: The FinSCDgen Study
Source: PLoS One. 2012 Jul 23;7(7):e41675. doi: 10.1371/journal.pone.0041675 (PMC3402479; doi:10.1371/journal.pone.0041675)
Supplement: Table S9 — SCD meta-analysis results for all studied SNPs. (PDF) [file pone.0041675.s010.pdf]

Table S9. SCD meta-analysis results for all studied SNPs.

SNP	Model	Population cohorts, HR (95% CI)				Autopsy studies, OR (95% CI)		Fixed-effects meta-analysis		
		FINRISK 1992	FINRISK 1997	FINRISK 2002	Health 2000	HSDS	TASTY	I^2	RR (95% CI)	*P*-value
rs846111	1	0.93 (0.70-1.24)	1.05 (0.82-1.33)	1.14 (0.79-1.64)	1.29 (0.97-1.73)	0.88 (0.58-1.32)	1.04 (0.73-1.47)	0.00	1.06 (0.93-1.20)	0.39
rs846111	2	0.96 (0.72-1.28)	1.06 (0.82-1.38)	1.25 (0.86-1.81)	1.37 (1.02-1.83)	NA	NA	0.11	1.13 (0.98-1.31)	0.10
rs846111	3	NA	1.11 (0.85-1.43)	1.26 (0.87-1.83)	1.42 (1.06-1.91)	NA	NA	0.00	1.24 (1.05-1.48)	0.01
rs846111	4	0.95 (0.71-1.27)	1.04 (0.80-1.35)	1.28 (0.88-1.85)	1.39 (1.04-1.86)	NA	NA	0.27	1.13 (0.98-1.31)	0.10
rs2880058	1	0.99 (0.77-1.28)	1.02 (0.82-1.28)	1.19 (0.85-1.65)	1.08 (0.83-1.42)	0.93 (0.65-1.32)	1.16 (0.82-1.66)	0.00	1.05 (0.94-1.18)	0.41
rs2880058	2	0.93 (0.72-1.21)	0.92 (0.72-1.17)	1.20 (0.86-1.68)	1.04 (0.78-1.37)	NA	NA	0.00	0.99 (0.87-1.14)	0.93
rs2880058	3	NA	0.90 (0.71-1.15)	1.19 (0.85-1.66)	1.03 (0.78-1.37)	NA	NA	0.00	1.01 (0.86-1.18)	0.96
rs2880058	4	0.93 (0.72-1.20)	0.93 (0.73-1.19)	1.24 (0.88-1.73)	1.01 (0.76-1.33)	NA	NA	0.00	0.99 (0.87-1.14)	0.91
rs12036340	1	0.95 (0.71-1.28)	1.02 (0.80-1.31)	1.36 (0.95-1.95)	1.29 (0.96-1.74)	0.92 (0.62-1.36)	1.20 (0.81-1.75)	0.00	1.10 (0.97-1.25)	0.15
rs12036340	2	0.88 (0.66-1.19)	0.87 (0.67-1.15)	1.37 (0.97-1.95)	1.24 (0.92-1.67)	NA	NA	0.54*	1.06 (0.85-1.32)	0.63
rs12036340	3	NA	0.86 (0.66-1.12)	1.35 (0.95-1.92)	1.23 (0.91-1.66)	NA	NA	0.60*	1.11 (0.84-1.46)	0.48
rs12036340	4	0.87 (0.65-1.18)	0.88 (0.67-1.15)	1.40 (0.98-1.99)	1.16 (0.86-1.56)	NA	NA	0.49	1.02 (0.88-1.19)	0.77
rs12143842	1	0.88 (0.65-1.18)	1.06 (0.83-1.36)	1.26 (0.88-1.81)	1.26 (0.95-1.68)	0.91 (0.61-1.34)	1.19 (0.81-1.74)	0.00	1.08 (0.95-1.23)	0.25
rs12143842	2	0.81 (0.60-1.09)	0.91 (0.69-1.19)	1.27 (0.89-1.81)	1.21 (0.91-1.62)	NA	NA	0.49	1.01 (0.87-1.18)	0.87
rs12143842	3	NA	0.89 (0.68-1.16)	1.25 (0.87-1.78)	1.20 (0.90-1.61)	NA	NA	0.37	1.07 (0.90-1.27)	0.44
rs12143842	4	0.80 (0.59-1.08)	0.91 (0.69-1.19)	1.30 (0.91-1.85)	1.12 (0.84-1.50)	NA	NA	0.43	0.99 (0.85-1.15)	0.90
rs10919071	1	0.94 (0.62-1.43)	0.88 (0.62-1.27)	1.48 (0.91-2.39)	1.12 (0.74-1.70)	0.91 (0.51-1.57)	0.80 (0.47-1.30)	0.00	1.00 (0.83-1.19)	0.96
rs10919071	2	0.98 (0.65-1.49)	0.82 (0.56-1.22)	1.43 (0.88-2.34)	1.11 (0.72-1.69)	NA	NA	0.05	1.03 (0.83-1.28)	0.77
rs10919071	3	NA	0.79 (0.53-1.17)	1.45 (0.89-2.36)	1.09 (0.71-1.66)	NA	NA	0.45	1.03 (0.80-1.32)	0.81
rs10919071	4	1.01 (0.67-1.54)	0.78 (0.52-1.15)	1.47 (0.90-2.40)	1.12 (0.74-1.71)	NA	NA	0.29	1.03 (0.83-1.28)	0.77
rs1805126	1	1.22 (0.95-1.57)	1.07 (0.87-1.33)	1.51 (1.08-2.11)	0.99 (0.76-1.30)	1.10 (0.78-1.55)	1.16 (0.82-1.64)	0.00	1.14 (1.02-1.28)	0.02
rs1805126	2	1.26 (0.98-1.61)	1.00 (0.79-1.25)	1.50 (1.07-2.09)	0.99 (0.75-1.31)	NA	NA	0.45	1.14 (1.00-1.30)	0.06
rs1805126	3	NA	0.96 (0.76-1.21)	1.51 (1.08-2.11)	1.02 (0.77-1.35)	NA	NA	0.61*	1.12 (0.86-1.44)	0.40
rs1805126	4	1.27 (0.99-1.63)	0.97 (0.77-1.23)	1.56 (1.12-2.17)	1.03 (0.78-1.37)	NA	NA	0.54*	1.17 (0.96-1.42)	0.13
rs12053903	1	1.28 (1.00-1.64)	1.04 (0.84-1.29)	1.38 (1.00-1.92)	0.98 (0.75-1.29)	1.12 (0.80-1.59)	1.06 (0.74-1.50)	0.00	1.12 (1.00-1.26)	0.045
rs12053903	2	1.32 (1.03-1.68)	1.04 (0.82-1.31)	1.37 (0.98-1.89)	0.98 (0.74-1.30)	NA	NA	0.28	1.15 (1.01-1.31)	0.041
rs12053903	3	NA	0.99 (0.78-1.26)	1.37 (0.99-1.90)	1.01 (0.76-1.34)	NA	NA	0.27	1.08 (0.92-1.26)	0.37
rs12053903	4	1.32 (1.03-1.69)	1.03 (0.81-1.30)	1.42 (1.03-1.97)	1.03 (0.77-1.37)	NA	NA	0.29	1.17 (1.02-1.34)	0.02

rs41312391	1	1.43 (1.09-1.87)	1.32 (1.04-1.68)	1.41 (0.97-2.04)	1.05 (0.76-1.46)	1.20 (0.78-1.83)	1.09 (0.72-1.62)	0.00	1.27 (1.11-1.45)	$3.4x10^{-4}$
rs41312391	2	1.53 (1.16-2.01)	1.33 (1.03-1.73)	1.44 (0.99-2.08)	1.07 (0.76-1.50)	NA	NA	0.00	1.35 (1.16-1.57)	$1.2x10^{-4}$
rs41312391	3	NA	1.24 (0.95-1.61)	1.44 (0.99-2.09)	1.10 (0.79-1.55)	NA	NA	0.00	1.24 (1.04-1.49)	0.02
rs41312391	4	1.55 (1.18-2.05)	1.28 (0.98-1.66)	1.47 (1.02-2.14)	1.16 (0.82-1.64)	NA	NA	0.00	1.36 (1.17-1.58)	$6.8x10^{-5}$
rs3922844	1	0.90 (0.68-1.20)	1.24 (0.98-1.56)	1.04 (0.71-1.52)	1.29 (0.96-1.73)	0.88 (0.58-1.33)	0.96 (0.67-1.38)	0.10	1.08 (0.95-1.23)	0.22
rs3922844	2	0.90 (0.67-1.22)	1.16 (0.90-1.48)	1.08 (0.74-1.58)	1.24 (0.92-1.68)	NA	NA	0.00	1.10 (0.95-1.27)	0.22
rs3922844	3	NA	1.16 (0.91-1.49)	1.09 (0.75-1.60)	1.25 (0.92-1.70)	NA	NA	0.00	1.18 (0.99-1.40)	0.07
rs3922844	4	0.90 (0.67-1.21)	1.12 (0.87-1.44)	1.09 (0.74-1.59)	1.20 (0.88-1.63)	NA	NA	0.00	1.07 (0.92-1.24)	0.37
rs6599219	1	0.91 (0.70-1.17)	1.01 (0.81-1.27)	1.09 (0.78-1.53)	0.80 (0.60-1.06)	1.01 (0.72-1.43)	1.05 (0.76-1.45)	0.00	0.97 (0.86-1.08)	0.54
rs6599219	2	0.97 (0.75-1.25)	1.06 (0.84-1.35)	1.04 (0.74-1.45)	0.79 (0.59-1.06)	NA	NA	0.00	0.97 (0.84-1.11)	0.62
rs6599219	3	NA	1.06 (0.83-1.35)	1.06 (0.75-1.48)	0.79 (0.59-1.05)	NA	NA	0.28	0.96 (0.82-1.13)	0.66
rs6599219	4	0.98 (0.76-1.26)	1.08 (0.85-1.38)	1.06 (0.75-1.49)	0.81 (0.61-1.09)	NA	NA	0.00	0.98 (0.86-1.13)	0.79
rs7372712	1	0.90 (0.63-1.27)	0.88 (0.65-1.20)	1.25 (0.83-1.89)	1.16 (0.83-1.62)	0.84 (0.50-1.41)	1.14 (0.76-1.69)	0.00	1.01 (0.87-1.18)	0.86
rs7372712	2	0.97 (0.67-1.40)	0.84 (0.61-1.18)	1.20 (0.79-1.84)	1.12 (0.80-1.58)	NA	NA	0.00	1.01 (0.84-1.21)	0.91
rs7372712	3	NA	0.90 (0.64-1.25)	1.23 (0.81-1.88)	1.10 (0.78-1.54)	NA	NA	0.00	1.04 (0.85-1.28)	0.69
rs7372712	4	0.97 (0.67-1.40)	0.88 (0.63-1.23)	1.23 (0.80-1.87)	1.12 (0.79-1.58)	NA	NA	0.00	1.02 (0.85-1.23)	0.81
rs2200733	1	1.15 (0.83-1.59)	1.15 (0.88-1.49)	1.55 (1.04-2.32)	1.42 (1.02-1.98)	1.12 (0.71-1.78)	1.58 (1.00-2.50)	0.00	1.28 (1.11-1.48)	$7.9x10^{-4}$
rs2200733	2	1.06 (0.77-1.47)	1.23 (0.93-1.63)	1.67 (1.11-2.50)	1.30 (0.92-1.82)	NA	NA	0.00	1.26 (1.07-1.49)	0.006
rs2200733	3	NA	1.20 (0.90-1.60)	1.60 (1.06-2.40)	1.28 (0.91-1.81)	NA	NA	0.00	1.31 (1.08-1.59)	0.006
rs2200733	4	1.03 (0.75-1.43)	1.18 (0.88-1.57)	1.53 (1.02-2.31)	1.19 (0.84-1.69)	NA	NA	0.00	1.19 (1.01-1.41)	0.040
rs2200733	5	NA	NA	NA	1.22 (0.85-1.74)	NA	NA	NA	NA	NA
rs10033464	1	1.28 (0.92-1.77)	0.92 (0.68-1.25)	1.19 (0.78-1.82)	0.94 (0.64-1.36)	1.32 (0.82-2.13)	1.20 (0.73-1.93)	0.00	1.10 (0.94-1.28)	0.23
rs10033464	2	1.36 (0.98-1.90)	0.91 (0.65-1.26)	1.23 (0.80-1.90)	1.03 (0.70-1.50)	NA	NA	0.09	1.11 (0.93-1.33)	0.27
rs10033464	3	NA	0.91 (0.66-1.27)	1.26 (0.82-1.95)	1.04 (0.71-1.51)	NA	NA	0.00	1.03 (0.83-1.28)	0.77
rs10033464	4	1.36 (0.97-1.90)	0.95 (0.68-1.32)	1.27 (0.82-1.97)	0.96 (0.65-1.41)	NA	NA	0.07	1.12 (0.93-1.34)	0.24
rs1042714	1	0.78 (0.60-1.01)	1.22 (0.99-1.51)	0.81 (0.58-1.14)	0.96 (0.73-1.27)	1.07 (0.75-1.54)	1.12 (0.80-1.57)	0.44	1.00 (0.89-1.12)	0.99
rs1042714	2	0.83 (0.64-1.07)	1.28 (1.01-1.61)	0.89 (0.63-1.25)	0.96 (0.73-1.28)	NA	NA	0.57*	0.99 (0.80-1.22)	0.90
rs1042714	3	NA	1.27 (1.01-1.60)	0.90 (0.64-1.26)	0.96 (0.72-1.27)	NA	NA	0.45	1.08 (0.92-1.26)	0.36
rs1042714	4	0.83 (0.64-1.08)	1.29 (1.03-1.63)	0.90 (0.64-1.26)	1.00 (0.76-1.33)	NA	NA	0.57*	1.01 (0.82-1.24)	0.96
rs12210810	1	0.79 (0.37-1.67)	0.77 (0.40-1.49)	1.77 (0.86-3.61)	1.04 (0.51-2.10)	0.52 (0.14-1.63)	0.68 (0.19-1.95)	0.00	0.94 (0.68-1.30)	0.71
rs12210810	2	0.89 (0.42-1.91)	0.77 (0.38-1.56)	1.60 (0.77-3.36)	0.89 (0.45-1.78)	NA	NA	0.00	0.99 (0.69-1.42)	0.94
rs12210810	3	NA	0.69 (0.34-1.42)	1.55 (0.73-3.27)	0.94 (0.47-1.87)	NA	NA	0.14	0.99 (0.65-1.50)	0.96

rs12210810	4	0.91 (0.43-1.94)	0.73 (0.36-1.49)	1.59 (0.76-3.34)	0.88 (0.44-1.78)	NA	NA	0.00	0.98 (0.68-1.40)	0.89
rs4725982	1	0.73 (0.54-0.97)	1.08 (0.86-1.36)	1.00 (0.69-1.43)	1.23 (0.92-1.65)	1.11 (0.76-1.62)	1.66 (1.15-2.39)	0.62*	1.09 (0.89-1.34)	0.42
rs4725982	2	0.62 (0.46-0.84)	1.08 (0.85-1.38)	0.98 (0.68-1.41)	1.24 (0.93-1.66)	NA	NA	0.75*	0.95 (0.71-1.28)	0.75
rs4725982	3	NA	1.05 (0.82-1.35)	0.97 (0.67-1.40)	1.23 (0.91-1.64)	NA	NA	0.00	1.09 (0.92-1.29)	0.32
rs4725982	4	0.60 (0.45-0.82)	1.11 (0.87-1.42)	0.97 (0.67-1.40)	1.19 (0.88-1.59)	NA	NA	0.76*	0.94 (0.69-1.28)	0.70
rs1805123	1	1.39 (1.04-1.88)	1.06 (0.80-1.40)	0.55 (0.31-0.95)	0.89 (0.62-1.26)	NA	NA	0.69*	0.97 (0.71-1.34)	0.87
rs1805123	2	1.42 (1.05-1.91)	1.03 (0.76-1.40)	0.58 (0.33-1.02)	0.83 (0.58-1.20)	NA	NA	0.69*	0.97 (0.70-1.34)	0.84
rs1805123	3	NA	1.08 (0.79-1.47)	0.58 (0.33-1.01)	0.83 (0.58-1.20)	NA	NA	0.49	0.90 (0.72-1.11)	0.32
rs1805123	4	1.48 (1.10-1.99)	1.03 (0.76-1.39)	0.59 (0.34-1.03)	0.82 (0.57-1.19)	NA	NA	0.72*	0.97 (0.69-1.37)	0.86
rs3807375	1	0.86 (0.67-1.11)	1.05 (0.85-1.29)	1.09 (0.79-1.52)	1.52 (1.15-2.00)	1.29 (0.92-1.82)	1.49 (1.07-2.11)	0.61*	1.18 (0.98-1.41)	0.08
rs3807375	2	0.77 (0.60-0.99)	1.01 (0.81-1.26)	1.07 (0.77-1.49)	1.52 (1.16-2.01)	NA	NA	0.77*	1.06 (0.80-1.39)	0.69
rs3807375	3	NA	1.02 (0.82-1.28)	1.07 (0.77-1.50)	1.50 (1.14-1.98)	NA	NA	0.59*	1.18 (0.92-1.51)	0.20
rs3807375	4	0.75 (0.58-0.97)	1.06 (0.85-1.32)	1.04 (0.74-1.45)	1.47 (1.11-1.93)	NA	NA	0.76*	1.05 (0.80-1.37)	0.75
rs2383207	1	1.04 (0.81-1.32)	1.00 (0.81-1.24)	1.15 (0.83-1.60)	1.06 (0.82-1.38)	1.31 (0.91-1.90)	1.61 (1.17-2.23)	0.31	1.13 (1.01-1.26)	0.04
rs2383207	2	0.98 (0.77-1.25)	0.89 (0.71-1.12)	1.08 (0.77-1.51)	1.02 (0.78-1.32)	NA	NA	0.00	0.98 (0.86-1.11)	0.71
rs2383207	3	NA	0.93 (0.74-1.18)	1.07 (0.77-1.50)	1.05 (0.80-1.36)	NA	NA	0.00	1.00 (0.86-1.17)	1.00
rs2383207	4	0.98 (0.77-1.25)	0.90 (0.71-1.14)	1.05 (0.75-1.48)	1.08 (0.83-1.41)	NA	NA	0.00	0.99 (0.87-1.13)	0.85
rs2074238	1	0.98 (0.65-1.47)	0.77 (0.52-1.15)	1.22 (0.72-2.07)	1.15 (0.76-1.74)	1.03 (0.57-1.84)	1.03 (0.55-1.89)	0.00	1.00 (0.82-1.21)	0.98
rs2074238	2	0.97 (0.64-1.48)	0.77 (0.50-1.18)	1.16 (0.68-1.96)	1.10 (0.73-1.65)	NA	NA	0.00	0.98 (0.78-1.22)	0.82
rs2074238	3	NA	0.82 (0.53-1.25)	1.17 (0.69-2.00)	1.13 (0.75-1.70)	NA	NA	0.00	1.01 (0.78-1.31)	0.92
rs2074238	4	0.97 (0.64-1.48)	0.84 (0.55-1.29)	1.22 (0.72-2.07)	1.13 (0.76-1.70)	NA	NA	0.00	1.02 (0.82-1.27)	0.89
rs757092	1	0.89 (0.69-1.15)	0.91 (0.72-1.14)	0.82 (0.58-1.17)	1.25 (0.96-1.64)	0.94 (0.65-1.36)	0.90 (0.62-1.30)	0.03	0.95 (0.85-1.07)	0.43
rs757092	2	0.79 (0.60-1.04)	0.87 (0.68-1.11)	0.81 (0.56-1.15)	1.17 (0.89-1.55)	NA	NA	0.37	0.90 (0.79-1.04)	0.15
rs757092	3	NA	0.88 (0.69-1.12)	0.80 (0.56-1.15)	1.17 (0.88-1.55)	NA	NA	0.41	0.95 (0.81-1.12)	0.52
rs757092	4	0.80 (0.61-1.05)	0.88 (0.70-1.13)	0.82 (0.58-1.18)	1.17 (0.88-1.55)	NA	NA	0.28	0.91 (0.79-1.05)	0.19
rs12576239	1	1.05 (0.75-1.47)	0.73 (0.53-1.01)	0.87 (0.55-1.37)	0.98 (0.67-1.42)	1.17 (0.73-1.87)	0.91 (0.57-1.41)	0.00	0.92 (0.79-1.08)	0.32
rs12576239	2	0.99 (0.71-1.39)	0.72 (0.51-1.01)	0.83 (0.52-1.32)	0.96 (0.66-1.40)	NA	NA	0.00	0.87 (0.72-1.05)	0.14
rs12576239	3	NA	0.77 (0.54-1.08)	0.83 (0.52-1.32)	0.94 (0.64-1.37)	NA	NA	0.00	0.84 (0.67-1.05)	0.12
rs12576239	4	0.98 (0.70-1.38)	0.74 (0.53-1.04)	0.86 (0.53-1.37)	0.90 (0.61-1.33)	NA	NA	0.00	0.87 (0.72-1.04)	0.13
rs10798	1	0.89 (0.69-1.16)	1.08 (0.86-1.35)	1.06 (0.76-1.49)	1.15 (0.88-1.52)	1.20 (0.85-1.72)	0.91 (0.64-1.29)	0.00	1.04 (0.93-1.17)	0.49
rs10798	2	0.89 (0.68-1.16)	1.08 (0.85-1.37)	1.05 (0.75-1.47)	1.13 (0.86-1.50)	NA	NA	0.00	1.03 (0.90-1.18)	0.67
rs10798	3	NA	1.07 (0.84-1.36)	1.05 (0.75-1.48)	1.12 (0.85-1.48)	NA	NA	0.00	1.08 (0.92-1.27)	0.33

rs10798	4	0.86 (0.66-1.12)	1.09 (0.86-1.39)	1.04 (0.74-1.45)	1.11 (0.83-1.47)	NA	NA	0.00	1.02 (0.89-1.17)	0.79
rs735951	1	0.81 (0.63-1.05)	1.06 (0.85-1.32)	1.00 (0.72-1.39)	0.95 (0.73-1.25)	0.95 (0.66-1.35)	1.00 (0.72-1.39)	0.00	0.96 (0.86-1.08)	0.50
rs735951	2	0.80 (0.61-1.04)	1.06 (0.84-1.34)	1.00 (0.71-1.40)	0.94 (0.72-1.24)	NA	NA	0.00	0.95 (0.83-1.09)	0.45
rs735951	3	NA	1.08 (0.85-1.36)	0.99 (0.71-1.39)	0.94 (0.71-1.24)	NA	NA	0.00	1.01 (0.86-1.19)	0.88
rs735951	4	0.79 (0.60-1.03)	1.10 (0.87-1.39)	1.02 (0.73-1.43)	0.98 (0.74-1.29)	NA	NA	0.16	0.97 (0.84-1.11)	0.62
rs37062	1	1.02 (0.77-1.34)	0.95 (0.74-1.20)	1.03 (0.72-1.49)	1.06 (0.78-1.45)	1.27 (0.85-1.90)	0.97 (0.66-1.43)	0.00	1.03 (0.90-1.16)	0.71
rs37062	2	1.06 (0.80-1.41)	0.94 (0.73-1.21)	0.96 (0.67-1.39)	1.05 (0.77-1.43)	NA	NA	0.00	1.00 (0.86-1.16)	0.99
rs37062	3	NA	0.95 (0.74-1.23)	0.98 (0.68-1.42)	1.06 (0.78-1.46)	NA	NA	0.00	0.99 (0.83-1.18)	0.92
rs37062	4	1.06 (0.79-1.41)	0.93 (0.72-1.20)	0.98 (0.68-1.42)	1.01 (0.74-1.37)	NA	NA	0.00	0.99 (0.85-1.15)	0.87
rs17779747	1	0.81 (0.60-1.09)	1.04 (0.82-1.32)	1.03 (0.71-1.49)	0.84 (0.62-1.15)	0.95 (0.62-1.43)	1.05 (0.74-1.47)	0.00	0.95 (0.83-1.08)	0.42
rs17779747	2	0.90 (0.67-1.22)	1.02 (0.79-1.32)	1.02 (0.70-1.48)	0.88 (0.64-1.20)	NA	NA	0.00	0.96 (0.82-1.11)	0.55
rs17779747	3	NA	1.04 (0.80-1.34)	0.99 (0.69-1.44)	0.86 (0.63-1.19)	NA	NA	0.00	0.97 (0.81-1.16)	0.74
rs17779747	4	0.91 (0.67-1.23)	1.02 (0.78-1.32)	0.97 (0.67-1.40)	0.87 (0.63-1.20)	NA	NA	0.00	0.95 (0.81-1.10)	0.47
rs1805128	1	0.89 (0.37-2.16)	0.71 (0.29-1.72)	0.89 (0.22-3.59)	1.11 (0.35-3.51)	0.52 (0.07-2.33)	1.00 (0.14-4.86)	0.00	0.84 (0.53-1.34)	0.46
rs1805128	2	0.93 (0.39-2.23)	0.71 (0.26-1.90)	1.00 (0.25-4.10)	1.06 (0.33-3.39)	NA	NA	0.00	0.89 (0.53-1.52)	0.68
rs1805128	3	NA	0.66 (0.24-1.78)	1.04 (0.25-4.25)	1.12 (0.35-3.57)	NA	NA	0.00	0.87 (0.45-1.69)	0.67
rs1805128	4	0.90 (0.37-2.17)	0.69 (0.25-1.85)	1.05 (0.26-4.30)	1.19 (0.37-3.78)	NA	NA	0.00	0.90 (0.53-1.53)	0.70
rs727957	1	0.93 (0.67-1.29)	0.93 (0.71-1.23)	0.89 (0.57-1.41)	0.71 (0.48-1.07)	0.96 (0.60-1.52)	1.11 (0.73-1.67)	0.00	0.92 (0.79-1.07)	0.26
rs727957	2	0.92 (0.66-1.29)	0.91 (0.67-1.23)	0.94 (0.59-1.51)	0.65 (0.43-0.98)	NA	NA	0.00	0.86 (0.72-1.03)	0.10
rs727957	3	NA	0.93 (0.68-1.25)	0.95 (0.59-1.51)	0.66 (0.44-1.01)	NA	NA	0.00	0.85 (0.69-1.06)	0.14
rs727957	4	0.91 (0.65-1.28)	0.93 (0.69-1.26)	0.96 (0.60-1.54)	0.66 (0.44-1.00)	NA	NA	0.00	0.87 (0.73-1.05)	0.14

Model 1: Age was used as the time scale and sex and geographic region were adjusted for. For HSDS and TASTY, age at death and sex were adjusted for. Model 2: Sex, geographic region, HDL-total cholesterol ratio, systolic blood pressure, prevalent diabetes, BMI, current and former smoking status, physical activity, and prevalent CHD were adjusted for. Model 3: Adjustments as in model 2 + QT-prolonging and QT-shortening drug use. Model 4: Adjustments as in model 2 + prevalent heart failure. Model 5 for rs2200733: Adjustments as in model 2 + atrial fibrillation. BMI = body mass index, CHD = coronary heart disease, CI = confidence interval, HDL = high-density lipoprotein cholesterol, HR = hazard ratio (in Cox regression), HSDS = The Helsinki Sudden Death Study, I^2 = measure of heterogeneity between studies, OR = odds ratio (in logistic regression), RR = relative risk, SNP = single nucleotide polymorphism, TASTY = The Tampere Autopsy Study.

*Random-effects meta-analysis was used because of significant heterogeneity between studies.
